# Perceptions of Mobile Health Apps and Features to Support Psychosocial Well-being Among Frontline Health Care Workers Involved in the COVID-19 Pandemic Response: Qualitative Study

**DOI:** 10.2196/26282

**Published:** 2021-05-31

**Authors:** Sungwon Yoon, Hendra Goh, Gayathri Devi Nadarajan, Sharon Sung, Irene Teo, Jungup Lee, Marcus E H Ong, Nicholas Graves, Tess Lin Teo

**Affiliations:** 1 Health Services and Systems Research Duke-NUS Medical School Singapore Singapore; 2 Department of Emergency Medicine Singapore General Hospital Singapore Singapore; 3 Lien Centre for Palliative Care Duke-NUS Medical School Singapore Singapore; 4 Department of Social Work Faculty of Arts and Social Sciences National University of Singapore Singapore Singapore

**Keywords:** COVID-19, frontline health care workers, mHealth, well-being, psychosocial

## Abstract

**Background:**

Frontline health care workers are experiencing a myriad of physical and psychosocial challenges amid the COVID-19 pandemic. There is growing recognition that digital technologies have the potential to improve the well-being of frontline workers. However, there has been limited development of wellness interventions using mobile health (mHealth) technology. More importantly, little research has been conducted on how frontline workers perceive mHealth-based support to promote their well-being.

**Objective:**

This study aimed to explore frontline workers’ experience of conventional psychological wellness programs and their perceptions of the usefulness of mHealth apps and features for promoting well-being. It also sought to identify factors that could potentially influence uptake and retention of an mHealth-based wellness program.

**Methods:**

We conducted semistructured interviews using purposive sampling with frontline workers involved in the COVID-19 response. Various visual materials, collated from existing mHealth app features, were presented to facilitate discussion. Interviews were audio-recorded and transcribed verbatim. Thematic analysis based on grounded theory was undertaken. Themes were subsequently mapped to key nudge strategies—those commonly used for mHealth development—to assess participants’ preferences for particular features and their reasoning.

**Results:**

A total of 42 frontline workers participated in 12 one-on-one interviews or focus group discussions. Frontline workers generally had a limited ability to identify their own psychological problems and liked the *reminders* functionality of the app to track their mood over time. A personalized goal-setting feature (ie, *tailoring*) and in-app *resources* were generally valued, while frequent coaching and messages (ie, *framing*) were seen as a distraction. The majority of participants desired a built-in chat function with a counselor (ie, *guidance*) for reasons of accessibility and protection of privacy. Very few participants appreciated a *gamification* function. Frontline workers commonly reported the need for ongoing social support and desired access to an in-app peer support community (ie, *social influence*). There were, however, concerns regarding potential risks from virtual peer interactions. Intrinsic motivational factors, mHealth app technicality, and tangible rewards were identified as critical for uptake and retention.

**Conclusions:**

Our study highlights the potential of mHealth apps with relevant features to be used as wellness tools by frontline health care workers. Future work should focus on developing a nonintrusive and personalized mHealth app with in-app counseling, peer support to improve well-being, and tangible and extrinsic rewards to foster continued use.

## Introduction

As the COVID-19 pandemic continues to claim more lives, health systems globally have been severely strained. The consequence has been mounting pressure on frontline health care workers [1]. Being at the heart of delivering essential health services during the pandemic, frontline workers face an increased burden and risk of developing physical and psychosocial problems [2]. Studies of previous infectious disease crises, such as SARS, H1N1, and Ebola, reported that frontline workers suffered from poor emotional and social functioning, depressive symptoms, and insomnia, all in addition to the distress relating to the risk of being infected and infecting their loved ones [3-5]. Likewise, emerging literature on COVID-19 consistently indicates a myriad of physical, psychological, and social challenges faced by frontline workers, including burnout, posttraumatic stress symptoms, anxiety, and depression [6,7].

Learning from the experience of prior public health emergencies, it has been suggested that support for the mental well-being of frontline workers is a requisite strategy to ensure a healthy and resilient workforce capable of providing a sustained COVID-19 response. Many institutions worldwide have begun to develop wellness intervention plans to support frontline workers during the pandemic [8-10]. These programs have included specialized mental care clinics, employee assistance counseling, and hospital-based interactive activities [11,12]. However, with few exceptions, the majority of the psychosocial support and wellness programs have been based on in-person interventions or one-off support, without much consideration given to sustained monitoring and impact assessment.

Organizations such as NHS (National Health Service) Digital in the United Kingdom and the US Department of Health and Human Services have underlined the importance of using mobile technology to improve access to care [13,14]. There is also a growing recognition that information and communication technologies have the potential to address the many challenges faced by the public and frontline workers during public health emergencies [15]. This technology focus has led to a surge in the development of mobile health (mHealth) apps during the ongoing COVID-19 pandemic [16,17]. According to recent review studies, a total of 29 mHealth apps pertaining to COVID-19 have been developed by 19 countries [18]. Yet, all of them were exclusively designed for contact tracing and symptom management with the primary purpose of containing disease transmission.

Despite increased rhetoric and enthusiasm for wellness support for frontline workers via virtual platforms, there remain limited advances toward developing psychosocial interventions using mHealth technology for this population. More importantly, little research so far has been undertaken on how frontline workers perceive mHealth-based support to meet their psychological needs and challenges during the ongoing COVID-19 situation. In light of the dearth of literature, this study aimed to explore frontline workers’ experience of conventional psychosocial wellness programs and their perceptions of the usefulness of mHealth apps and various features for promoting well-being. The study also sought to identify factors that could potentially influence uptake and retention of mHealth-based wellness support programs. Exploring frontline workers’ perspectives as end users will inform future efforts to design evidence-based and user-centered mHealth interventions.

In this study, we adopted nudge theory as a way to better understand perceptions of mHealth app features designed to support psychosocial well-being. Nudge theory is a novel concept that affects the process of decision making in individuals through choice architecture [19]. The theory has been widely used to develop mHealth apps in general and has also been applied to interventions promoting mental well-being. A growing body of literature has demonstrated evidence of nudge elements that promote health behavior and optimize user outcomes with varying degrees of effectiveness in mHealth interventions [20]. For example, a study by Wiecek et al highlighted that use of *reminders* is a successful intervention component for mHealth apps to improve medication adherence, as participants are regularly “nudged” to take their medication [21]. Martin et al called for the incorporation of *gamification* in an mHealth app, as it motivates the user to adopt intended behavior through gamified activities [22]. *Social influence* has also been considered an important nudge that increases the effects of intervention outcomes by fostering a feeling of belonging to a community with a common goal [23]. Individualized *tailoring* has been found to be an effective nudge in sustaining user engagement for digital mental health interventions [24-26]. In addition, provision of app-based resources yielded a positive outcome that alleviated chronic stress of working women [27]. Taken together, nudge theory has proved to be useful in effecting behavior change for mHealth interventions. Ahead of the design of an intervention to support the well-being of frontline workers, we sought to explore their views and preferences for app features that underpin these key nudge strategies.

## Methods

### Setting and Participants

This qualitative study was conducted in Singapore during the peak of the COVID-19 outbreak from April to August 2020. Singapore is a multiethnic city-state in Southeast Asia. Although the country’s public health care system consists of a team of highly trained health care professionals and efficient services, the surge in COVID-19 infections placed considerable strains on the health system [28]. Mass outbreaks in foreign worker dormitories in April 2020 spurred the government’s decision to impose a 2-month lockdown [29]. The evolving outbreak situation posed unprecedented risks that could compromise the well-being of frontline workers. In response, a multidisciplinary task force of the Singapore General Hospital, the largest public hospital in Singapore, swiftly rolled out a wellness program aimed at mitigating the risk of burnout and adverse stress reactions among frontline workers. The program included operational and psychological education, provision of a written handout for family members, active support of staff who were involved in high-risk areas, and close communication between peer support leaders and clinical team members.

It is against this background that a study was conducted to explore perceptions of frontline workers (ie, doctors and nurses) about mHealth apps to improve their well-being. The study was introduced to clinical teams at the Singapore General Hospital and Community Care Facilities—large-scale institutional isolation units—at their staff meetings. Participants were identified using official websites, the study team’s professional networks, and recommendations from other study participants. In this study, we defined frontline workers as those who were (1) doctors and nurses and (2) in direct contact with suspected and confirmed COVID-19 patients and provided essential health care services. Potential participants were invited to participate by email and were provided with background information. We used a purposive sampling approach to maximize the diversity of experiences and opinions. As the data collection and concurrent analyses progressed, the variation in emergent themes was explored by recruiting subsequent participants for focus groups and interviews based on profession and years of experience to improve our understanding of specific aspects of the studied phenomenon. Prior to interviews, informed consent was sought via email.

### Data Collection

A semistructured interview guide was developed based on relevant literature and the study team’s expert knowledge [30-32]. Major topics included perceptions of the existing wellness program available for frontline workers, perceived usefulness of mHealth apps for mental well-being, features that might be valuable for improving wellness, and factors affecting adoption of mHealth apps for wellness. In addition, self-reported demographic information, such as age, gender, ethnicity, and years of experience in health care, were collected. During the interview, various visual materials, collated from existing mHealth app features for wellness promotion, were presented to facilitate the discussion. These materials were organized according to key nudge strategies commonly used for mHealth app development [33,34]. They included, but were not limited to, the following strategies: framing, tailoring, reminders, social influence, guidance, social modeling, gamification, and resources [19]. These strategies enabled a structured way to collect participants’ perspectives on the usefulness of mHealth features for improving well-being. Due to the constraints of participants’ working hours, consented individuals took part in either a one-on-one interview or focus group discussion, subject to availability. For safety reasons, all interviews were conducted virtually over Zoom by two interviewers trained in qualitative research (SY and HG). Reflections and memos were written after each interview to capture insights. Interviews lasted 31 to 65 minutes. This study was approved by the National University of Singapore Institutional Review Board (NUSIRB No: S-20-115)

### Data Analysis

All interviews were audio-recorded following consent and were transcribed verbatim. Thematic data analysis was undertaken using grounded theory. A grounded theory approach based on the work of Glaser and Strauss was chosen because it explored experiential aspects of psychosocial programs and how participants perceived the usefulness of key features in a wellness app. The grounded theory approach allowed for emerging constructs and themes through iterations of data collection and analysis [35,36]. Two independent coders (SY and HG) reviewed the interview materials, summarized and extracted meaningful statements, and carried out open coding and axial coding using NVivo 12 (QSR International), a qualitative data analysis software. During open coding, transcripts were analyzed to develop categories of information. This allowed for subthemes to be derived from the data instead of pre-existing ideas. During axial coding, common subthemes were grouped into unifying themes. The iterative process of independent coding and consensus meetings continued until no new emergent themes were identified. The codes were independently applied to all transcripts, and coding discrepancies were resolved by iterative discussions. Lastly, for mHealth features, themes were mapped against the nudge strategies to assess participants’ preferences for particular features and their reasoning [34]. For rigor and transparency, we anchored our methodology according to the Consolidated Criteria for Reporting Qualitative Research (COREQ) checklist (Multimedia Appendix 1) [37].

## Results

### Characteristics of Participants

A total of 42 frontline workers participated in 12 one-on-one interviews or focus group discussions. Two one-on-one interviews and 10 focus group discussions were conducted, comprising 21 doctors and 21 nurses (for composition of participants in each session, see Table S1 in Multimedia Appendix 2). The recruitment rate was 93% (42/45). A total of 3 individuals out of 45 (7%) declined participation for reasons of lack of time and disinterest. During the peak of the COVID-19 outbreak, health care workers from a variety of clinical departments had been deployed to frontline clinical duties. Participants’ clinical home departments ranged across anesthesiology, dentistry, community nursing, and cardiology, among others. Data saturation was reached after the 10th interview, with no new themes emerging from subsequent interviews. We conducted two additional focus groups beyond data saturation to ensure that the point of information redundancy had been achieved. Table 1 shows the characteristics of participants, including 21 (50%) doctors and 21 (50%) nurses out of 42; 76% (32/42) of participants were female and 64% (27/42) were Chinese. The mean age of the sample was 29.6 (SD 3.9) years, with slightly more than half of the participants (24/42, 57%) aged 30 years and below. Participants’ experience in the health care sector ranged from 2 to 18 years.

**Table 1:** Characteristics of participants.

Characteristic			Value (N=42)
**Age (years)**			
	Mean (SD)		29.6 (3.9)
	**Category, n (%)**		
		<30	24 (57)
		30-50	18 (43)
**Ethnicity, n (%)**			
	Chinese		27 (64)
	Malay		7 (17)
	Indian		5 (12)
	Others		3 (7)
**Gender, n (%)**			
	Female		32 (76)
	Male		10 (24)
**Profession, n (%)**			
	Doctor		21 (50)
	Nurse		21 (50)
Years of experience in health care, mean (SD)			6.8 (3.6)

### Experience and Perceptions of the Conventional Psychosocial Program

Table 2 shows the experience of participants regarding the existing wellness program and their coping strategies. By and large, the wellness program was perceived to be appropriate, with some participants expressing positive thoughts about the educational nature of the session. In particular, junior doctors who had never experienced an outbreak response appreciated the opportunity to learn how to protect themselves and where to find resources in the event of burnout. However, as the program comprised a one-off session delivered to a mixture of different health care teams, some expressed the need for building rapport and relationship to enhance the experience. As one participant noted, the session was felt to be a “random group meeting with strangers” (Participant #19, doctor). A minority of participants were not aware of the program’s existence. The inherent absence of team spirit and social connectivity, coupled with limited awareness, seemed to discourage frontline workers from active involvement. Most participants preferred that the program session be organized according to clinical teams.

A recurring theme was that informal help-seeking appeared to be the most common avenue to ameliorate the emotional “fallout” from work for our participants. The majority indicated seeking support from family and friends when there was “emotional exhaustion.” This was more apparent for participants who had limited awareness of the formal support program or felt that the current program did not meet their expectations. Importantly, participants commonly noted that they often could not recognize their own symptoms of burnout. The cumulative psychological effects of burnout at times unwittingly resulted in high anger expression and feelings of frustration. When prompted, some participants cited constant fatigue and physical exhaustion amid longer work hours as the main reason that limited their ability to self-identify psycho-emotional signs and symptoms.

**Table 2:** Perception of in-person wellness program and personal coping practice.

Theme and subthemes			Illustrative quotes
**Experience of formal wellness program**			
	Greater need for building rapport and relationships	“I think the program was okay. But the group was huge, so I think sometimes people might be a bit shy to share feelings in a large group.” (Participant #1, doctor) “The social workers gave us their contact details after the session, which was good as we can call whenever we need help. But I feel some people might not utilize them as there was no prior rapport built.” (Participant #35, nurse)	
	Limited awareness of wellness program for uptake	“I was not aware of that [wellness program], but I do know that there are such services for the COVID-19 patients.” (Participant #11, doctor) “I don’t think there was a [wellness program], maybe I missed the email. Not too sure about that.” (Participant #42, doctor)	
	Lacking team spirit and social connectivity among colleagues	“The session was slightly awkward as it was just out of the blue. It was like a random group meeting with strangers, I feel.” (Participant #19, nurse) “It might be better to split the sessions into participants who are from the same team, as some people might be uncomfortable sharing in the presence of people whom they might not know.” (Participant #1, doctor)	
**Personal coping practice**			
	Informal help-seeking or non–help-seeking as a prevailing practice	“Let’s say on that particular day if I am feeling frustrated regarding work, I will simply talk to my family members, and they are very supportive.” (Participant #4, doctor) “I am sure the hospital has put in place some sort of program to help us psychologically, just that I never actively sought for help.” (Participant #17, doctor)	
	Limited ability to self-identify psycho-emotional symptoms	“Sometimes when I am so tired, I just don’t know what to feel anymore.” (Participant #39, nurse) “There was this time when I had a mini rage as everything just built up. Until a senior noticed this and she came over to ask me what happened and helped me to resolve the issues.” (Participant #14, doctor)	

### Perceived Usefulness of Features in Wellness mHealth Apps

Table 3 presents perceived usefulness of various features for an mHealth app. Across seven nudge strategies (ie, reminders, tailoring, guidance, framing, social influence or modeling, gamification, and resources), 14 themes emerged. It was commonly perceived that using *reminders* to nudge individuals to monitor their own psychological well-being would be helpful in improving self-awareness, particularly when tracking was visualized in a graphic form for easy interpretation. As one participant noted, the tracking feature would allow for “reflection on stressors” (Participant #4, doctor) by alerting users of the presence of mood deterioration. However, frequent notifications were seen as an “annoyance.” In addition to the tracking feature, participants valued a personalized goal-setting feature (ie, *tailoring*), which could aid them by altering them to diet and sleep patterns. Almost all the participants desired a built-in chat with a counselor (ie, *guidance*), as it would offer greater convenience and enable ready access to professional care compared to the conventional mode of in-person counseling. However, many participants did not appreciate the artificial intelligence–driven chatbot, due to the loss of human interactivity. They expressed hesitance toward this feature, although some recognized that it might be useful for simple tasks such as requests for mental health resources.

To enhance the user experience, the acceptability of a *framing* feature was explored. Given frequent changes in work protocols in light of the COVID-19 situation and its consequential work demands, occasional messages and coaching were favored compared to daily push notifications, which were seen as an “annoyance.” Participants felt that the former would offer a sense of human touch without causing intrusion into private life. Under the *social influence* nudge strategy, features such as forum chats and in-app peer support groups were seen as an essential component of psychosocial support. Despite the positive perceptions of the social influence features, participants highlighted that proper safeguarding measures should be in place to ensure appropriate balance between freedom of expression and emotional manipulation, especially by users who post malicious comments. Related to this, some participants had concerns about disclosure of their personal well-being state that might inadvertently affect assessment of their work performance. This concern was more salient among junior doctors.

The *gamification* function, a widely used nudge strategy to improve self-efficacy, was not something participants were keen to use. While a minority saw a gaming strategy as entertainment, most believed that competition via scoring may be demotivating and backfire when individuals fall behind their peers and colleagues. Virtual rewards such as earning badges, intended for triggering competitive natures, were generally received in a negative light. App-based *resources*, such as mindfulness-based exercises and short wellness articles, were highly valued as a handy and useful tool in promoting a healthier lifestyle and mental well-being.

**Table 3:** Perceived usefulness of features in wellness mobile health apps.

Nudge strategy and categories			Theme		Illustrative quotes
**Reminders (ie, timely cues)**					
	Mood monitoring	Regular in-app tracking of a mood trend perceived as a tool for improving self-awareness of emotions		“I think the feature can help me to record when is the day that I am feeling low or happy.” (Participant #2, doctor)	
	Progress tracking	Regular in-app tracking of a mood trend perceived as a tool for improving self-awareness of emotions		“You can keep track of your progress and monitor your mood. Eventually, you can see a trend which might potentially help you to identify stressors in daily life. I won’t mind using it.” (Participant #5, doctor)	
	Push notification for engagement	Frequent notifications being felt as a distraction		“I do not like this idea [periodical receipt of push notifications], as I find it [to be] annoying. But as long as there is this option of turning it off, then it is fine.” (Participant #11, doctor)	
**Tailoring (ie, context-sensing based on input)**					
	Tailored feedback	Feedback system aids users in making beneficial changes		“I think this feature [feedback] is a good thing. I wouldn’t mind using it, as it allows me to understand better where I have done great and in which area I can work to improve my mental well-being.” (Participant #3, doctor)	
	Personalized goal setting	Setting personalized goals are perceived to be improving general well-being		“I think being able to set goals, like exercise for half an hour a day or sleep before 11 PM, might promote healthy living.” (Participant #18, doctor)	
**Guidance (ie, provision of practical advice)**					
	Artificial intelligence–based chatbot	Loss of human contact for emotional support is deemed meaningless		“If you are having some emotional issues, you probably wouldn’t want to talk to a robot who might not even understand your question properly.” (Participant #21, nurse)	
	Chat with counselor	In-app counseling providing greater convenience and access to care compared to conventional mode of interaction		“I think the chat with a counselor might be helpful, as it is pretty hard to get an appointment with them. And with the built-in feature, one can get a response almost immediately.” (Participant #17, doctor)	
	Chat with counselor	Enabling anonymous care		“I think we do a lot of texting nowadays, so I think texting with a counselor might help, especially if the counselor does not know our identity. So privacy is guaranteed while getting your problems sorted out with a professional.” (Participant #34, nurse)	
**Framing (ie, shaping of information that alters its perceived nature)**					
	Personalized messages	Personalized messages offer a sense of human touch		“A message like this [personalized message] might encourage me to check on myself more frequently. It feels like someone is concerned and telling me to take care of myself.” (Participant #12, doctor)	
**Social influence (ie, social support and conforming to a social trend)**					
	Forum	Emphasis on anonymity for concerns about risk of emotional manipulation and privacy		“Although it may be useful for sharing experiences and support one another, we will definitely need moderators to watch out for potential bullying or manipulation on the forum. In a support group, you can still have other people playing the [role] as a whistleblower, but for an anonymized forum chat, there may be someone who will keep posting malicious comments.” (Participant #31, nurse)	
	App-based peer support group	Peer support perceived as useful as a platform for seeking comfort and sharing experience with colleagues		“For people who require emotional support but choose not to share their problems in a face-to-face format, I think this is a handy feature to have.” (Participant #26, nurse)	
**Gamification (ie, tapping into an individual’s desire to progress in a game setting)**					
	Interactive game activities	Reservations about the effectiveness of game elements in improving wellness		“I think it might motivate people who like to play games or have the habit of playing games regularly to use the app. But it might not appeal to people who do not play games.” (Participant #40, doctor)	
	Scoring system	Comparing scores between users may discourage usage		“Personally, I feel that comparing points [between users] will only add to the stress, because people become competitive.” (Participant #27, nurse)	
**App-based resources**					
	Mindfulness-based exercises	In-app tutorials and short readings perceived as handy and useful		“This [mindfulness-based exercise] is very useful. It can potentially help me destress myself after my shift and sleep well.” (Participant #29, nurse)	
	Self-help aids (eg, short articles and videos)	In-app tutorials and short readings perceived as handy and useful		“Some people may want to look for useful articles, and if the articles are provided in the app itself, it will be really convenient and can potentially benefit a lot of people. It would be good if they are [presented] in point form.” (Participant #40, doctor)	

### Factors That Could Affect Adoption and Sustained Use of a Wellness mHealth App

Factors that could potentially influence the adoption of a wellness mHealth app among frontline health care workers are presented in Table 4. Three overarching factors and nine themes were identified: (1) technical factors, with the themes perceived ease of use, convenience, security, and information technology (IT) support; (2) personal factors, with the themes perceived usefulness, perceived vulnerability, and awareness; and (3) external factors, with the themes rewards and price of app. Participants noted that nonintrusiveness with a minimum set of features relevant to the needs of frontline workers would be of prime importance in prompting adoption and ensuring continued use. As one participant noted, a simple interface that requires “minimum manual input” (Participant #12, doctor) was one of the most desired features for continued use. A robust IT and security system was also brought up by participants to help them start or continue using an mHealth app.

**Figure 1:**
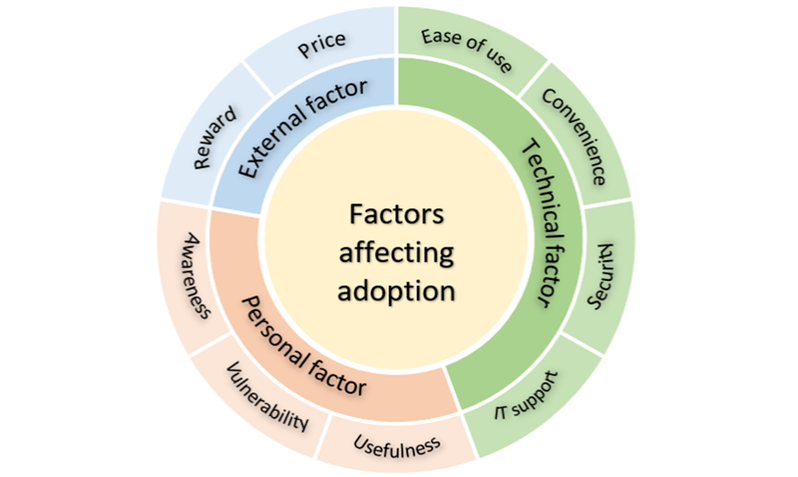
Factors affecting adoption and sustained use of a mobile health app. IT: information technology.

A few participants did not consider an mHealth app as necessary for promoting their mental health and wellness, mainly because they felt that they were coping well. Nonetheless, they thought that such an app might be helpful for colleagues who require psycho-emotional support or who are keen to improve their well-being. Increasing awareness of the availability of an app would be vital to its adoption. To encourage and sustain usage, affordable pricing of the app and attractive rewards could be considered to motivate users. Cost was one of the determining factors for app adoption: the majority of participants favored a free app or were willing to pay a small amount (eg, US $10) if it lived up to their expectations. More tangible and extrinsic rewards, such as Continuing Professional Education (CPE) points and taxi credits after accruing virtual points for adherence, were also suggested as motivation to sustain usage. Figure 1 summarizes the factors that may influence adoption and sustainability of a wellness mHealth app for frontline workers.

**Table 4:** Factors affecting potential adoption and sustained use of wellness mobile health (mHealth) apps.

Factors and themes			Illustrative quotes
**Technical factors**			
	Perceived ease of use	“I will use the app if it is easy to use. I think judicious use of color might catch people’s attention.” (Participant #12, doctor)	
	Convenience	“Because we are always using the phone, if you are feeling down, at least you can use the app to help you manage your emotions. I think it is quite convenient and handy.” (Participant #20, nurse)	
	Security and privacy	“A moderator has to be present to watch out for potential bullying and malicious comments [in forum chats] so as to make people feel safe.” (Participant #34, nurse)	
	Information technology (IT) support	“It will be good if there is a team of dedicated IT personnel to ensure the app is running well and to minimize the potential [hacking] and leaking of information.” (Participant #34, nurse)	
**Personal factors**			
	Tangible benefits	“I guess for the general health care workers, it [the app] might be useful in helping them to manage their emotions better, as there are many features that I feel are quite useful, such as the virtual counseling service and peer support.” (Participant #41, doctor)	
	Perceived vulnerability to wellness risks	“I might not use the app routinely, as I have been coping well, but for some colleagues, I can tell they are struggling with burnout. If the app is introduced to them, I feel they are likely to use it.” (Participant #19, nurse)	
	Awareness of app	“Many people did not participate in the wellness program, simply because they were not aware or did not feel the need to. Same thing for the app; it has to be made known to us before we can decide to use it.” (Participant #26, nurse)	
**External factors**			
	Rewards and incentives	“I think you need some rewards to encourage usage, apart from CPE [Continuing Professional Education] points; I think it will be great if rewards like grocery vouchers and taxi credits can be exchanged with the points we have accumulated in the app.” (Participant #21, nurse)	
	Cost	“I stopped using [previous mHealth app] because they wanted to charge a lot. So, if your app is free, then it will be wonderful. I will totally use it!” (Participant #5, doctor)	

## Discussion

### Principal Findings

This study sought to build on limited literature by exploring the perceived usefulness of mHealth apps and features to improve psychosocial well-being from the perspectives of frontline workers responding to the COVID-19 pandemic. Previous studies have suggested that psychological interventions could help frontline workers cope with emotional exhaustion and could ameliorate work-induced stress during public health emergencies [8,38,39] and that counseling reduces the risk of depression in frontline workers [40]. However, our study found that our current in-person counseling has limitations in its ability to monitor and manage physical and mental health issues faced by frontline workers. Our participants, who are working on the front line, commonly reported that lack of rapport and limited continuity and awareness of the program inhibited uptake and appreciation of in-person counseling and psycho-emotional education. This finding from our study reinforces that from a previous study that insufficient rapport-building led to diminished utilization of wellness support services among frontline workers [31]. These factors, together, underscore the importance of developing an optimal set of psychosocial interventions that address pertinent concerns and needs of frontline workers. Delivery of psychological interventions through digital devices can possibly help in overcoming some of the barriers identified, with the potential for wide implementation in a convenient, efficient, and cost-effective manner [41]. Current evidence indicates that mHealth-based interventions increased accessibility to counseling and psycho-education and improved resilience among health care workers [42,43]. Leveraging digital platforms to provide wellness support to frontline workers may be a viable approach. Consistent with previous research, frontline workers generally had limited ability to recognize their psychological problems [44]. Constant exposure to a high-intensity work environment and chronic fatigue appeared to impair the participants’ ability to recognize their psycho-emotional signs and symptoms. Hence, assisting frontline workers to identify their symptoms early in the process could be an important strategy for mitigating the risk of developing severe mental health issues. To this end, it is encouraging to note that frontline workers in our study desired a *reminder* functionality, such as tracking of mood and psychological stress, to understand their own mental health states. Another mHealth app feature that was highly valued by the frontline workers was in-app *resources*. This finding is consistent with evidence from previous studies indicating that reminders in mHealth apps played a vital role in improving emotional well-being. For instance, regular monitoring of mood was found to be associated with timely detection and mitigation of potential stressors [45]. Routine in-app reporting of thoughts and feelings can increase emotional self-awareness, which in turn, fosters coping skills [46]. A recent randomized controlled trial demonstrated that mood tracking resulted in improved coping ability, better adaptive response, and enhanced self-care [47]. Likewise, in-app resources, such as meditation and mindfulness exercises, were found to have a positive effect on improving subjective well-being and behavioral regulation [48]. Therefore, future mHealth interventions should incorporate key nudge strategies that are favored by frontline workers into the design to increase effectiveness.

It has been well-recognized that social support, such as being esteemed, valued, and part of a social network of mutual assistance, is strongly associated with improved mental health [49-51]. Perceived availability of social support may bolster one’s ability to cope with challenges, providing an avenue for emotional expression. Our study found that informal help-seeking from family and friends or non–help-seeking (ie, ignoring symptoms) were prevailing practices for frontline workers in response to emotional exhaustion associated with COVID-19. Frontline workers commonly reported the need for ongoing social support and desired an mHealth app with features that would enable them to readily access and check in with a virtual peer support community. Indeed, having similar experiences during the pandemic, peer health care workers may be in a unique position to provide practical and emotional support. A systematic review showed that digital peer support interventions led to improved self-coping and adaptation [27]. Through candid sharing of experiences and validation of feelings, app-based peer support, embedded in a *social influence* nudge strategy, could complement existing in-person support and have a positive impact on frontline workers’ psychological well-being [52]. There was, however, a common concern among our participants regarding potential risks arising from virtual peer interactions, such as the spread of misleading information, derogatory comments, and disclosure of personal mental health issues. Therefore, an appropriate safeguarding mechanism should be in place to ensure the best possible outcomes for frontline workers.

Despite several mHealth features that were valued by frontline workers, a crucial issue remains to be considered: adoption and sustained engagement. A recent study reported that nearly 70% of users abandoned mHealth apps after a single use or stopped using the apps after a month [53]. This trend could stem from various factors. Participants in our study reported that mHealth app technicality and intrinsic factors were critical for uptake and retention. This finding resonates with prior research [54] and highlights that a simple user interface would be one of the most important aspects of an mHealth wellness program for frontline workers. Communicating the benefits and relevance of the wellness app, as well as transparency of data management, would be equally important for the successful implementation of an mHealth app [55,56]. In contrast to prior literature, frontline workers were more inclined to engage with a wellness app if external incentives, such as CPE points, were offered [57]. Therefore, it may be helpful to consider incorporating app features that can be manipulated to optimize incentive effectiveness (ie, type of incentives, timing, and magnitude) to maintain engagement of frontline workers.

### Strengths and Limitations

This study added important evidence to the potential for wellness interventions delivered through an mHealth app from the perspectives of frontline health care workers. Findings from this study could provide valuable insight into the development and implementation of mHealth apps for improving the well-being of frontline workers involved in the response to COVID-19. Notwithstanding its strengths, this study was limited in several aspects. The results of this study were derived from qualitative research alone, which is, by nature, prone to a degree of potential subjectivity. Despite our efforts to recruit a balanced mix of genders, almost three-quarters of the participants were female. This might have introduced potential selection bias into the study. However, we did not find any considerable gender differences in viewpoints on key topics of interest. The discussion on mHealth features during the interviews was primarily based on visual materials underpinned by nudge strategies. Thus, participants’ responses might have been influenced by the mHealth features presented. Lastly, the study participants were limited to doctors and nurses; further research is needed to explore perceptions of mHealth app features to support well-being among administrative and ancillary staff as well as allied health care professionals working on the front line.

### Conclusions

Emerging technologies hold considerable promise for significantly expanding the reach of wellness programs for frontline health care workers. Traditional face-to-face modes of episodic support in times of public health emergencies seem to have limited utility to monitor and address the needs of frontline workers in a timely and holistic manner. Our study highlighted the need to take into account frontline workers’ preferences and values when designing mHealth-based models of care to promote their well-being. Future work should focus on developing a nonintrusive and personalized app with in-app counseling and peer support features to improve well-being as well as tangible and extrinsic rewards to foster continued use.

## Appendix

Multimedia Appendix 1 Consolidated Criteria for Reporting Qualitative Research (COREQ) checklist.

Multimedia Appendix 2 Composition of participants in each session of one-on-one interviews and focus group discussions.

## Abbreviations

COREQ: Consolidated Criteria for Reporting Qualitative Research
CPE: Continuing Professional Education
IT: information technology
mHealth: mobile health
NHS: National Health Service
