# Supplementary material for: Perceptions of Mobile Health Apps and Features to Support Psychosocial Well-being Among Frontline Health Care Workers Involved in the COVID-19 Pandemic Response: Qualitative Study
Source: J Med Internet Res. 2021 May 31;23(5):e26282. doi: 10.2196/26282 (PMC8168635; doi:10.2196/26282)
Supplement: Multimedia Appendix 2 [file jmir_v23i5e26282_app2.docx]

| **Session** | **Participant number** | **Gender** | **Profession** | **Mode of interview** |
| --- | --- | --- | --- | --- |
| 1 | 1 | F | Doctor | FGD |
|  | 2 | M |  |  |
| 2 | 3 | M | Doctor | FGD |
|  | 4 | M |  |  |
|  | 5 | F |  |  |
|  | 6 | F |  |  |
|  | 7 | F |  |  |
|  | 8 | F |  |  |
|  | 9 | F |  |  |
|  | 10 | F |  |  |
|  | 11 | M |  |  |
| 3 | 12 | F | Doctor | FGD |
|  | 13 | M |  |  |
|  | 14 | F |  |  |
|  | 15 | M |  |  |
| 4 | 16 | M | Doctor | FGD |
|  | 17 | F |  |  |
|  | 18 | F |  |  |
| 5 | 19 | F | Nurse | FGD |
|  | 20 | F |  |  |
|  | 21 | F |  |  |
| 6 | 22 | F | Nurse | FGD |
|  | 23 | F |  |  |
|  | 24 | M |  |  |
|  | 25 | F |  |  |
| 7 | 26 | F | Nurse | IDI |
| 8 | 27 | F | Nurse | FGD |
|  | 28 | F |  |  |
|  | 29 | F |  |  |
| 9 | 30 | F | Nurse | FGD |
|  | 31 | F |  |  |
|  | 32 | F |  |  |
|  | 33 | F |  |  |
| 10 | 34 | M | Nurse | FGD |
|  | 35 | F |  |  |
|  | 36 | F |  |  |
|  | 37 | F |  |  |
|  | 38 | F |  |  |
| 11 | 39 | F | Nurse | IDI |
| 12 | 40 | F | Doctor | FGD |
|  | 41 | M |  |  |
|  | 42 | F |  |  |

Supplementary Table S1. Focus group and in-depth interviews
